# Loss of the Phenolic Hydroxyl Group and Aromaticity from the Side Chain of Anti-Proliferative 10-Methyl-aplog-1, a Simplified Analog of Aplysiatoxin, Enhances Its Tumor-Promoting and Proinflammatory Activities

**DOI:** 10.3390/molecules22040631

**Published:** 2017-04-13

**Authors:** Yusuke Hanaki, Masayuki Kikumori, Harukuni Tokuda, Mutsumi Okamura, Shingo Dan, Naoko Adachi, Naoaki Saito, Ryo C. Yanagita, Kazuhiro Irie

**Affiliations:** 1 Division of Food Science and Biotechnology, Graduate School of Agriculture, Kyoto University, Kyoto 606-8502, Japan; hanaki.yuusuke.53s@st.kyoto-u.ac.jp (Y.H.); k9morin@gmail.com (M.K.); htokuda@med.kanazawa-u.ac.jp (H.T.); 2 Division of Molecular Pharmacology, Cancer Chemotherapy Center, Japanese Foundation for Cancer Research, Tokyo 135-8550, Japan; mutsumi.okamura@jfcr.or.jp (M.O.); sdan@jfcr.or.jp (S.D.); 3 Laboratory of Molecular Pharmacology, Biosignal Research Center, Kobe University, Kobe 657-8501, Japan; na@gold.kobe-u.ac.jp (N.A.); naosaito@kobe-u.ac.jp (N.S.); 4 Department of Applied Biological Science, Faculty of Agriculture, Kagawa University, Kagawa 761-0795, Japan; charlesy@ag.kagawa-u.ac.jp

**Keywords:** protein kinase C, PKC, C1 domain, aplysiatoxin, tumor-promoter, anti-cancer

## Abstract

Aplysiatoxin (ATX) is a protein kinase C (PKC) activator with potent tumor-promoting activity. In contrast, 10-methyl-aplog-1 (**1**), a simplified analog of ATX, was anti-proliferative towards several cancer cell lines without significant tumor-promoting and proinflammatory activities. To determine the effects of the phenolic group on the biological activities of **1**, we synthesized new derivatives (**2**, **3**) that lack the phenolic hydroxyl group and/or the aromatic ring. Compound **2**, like **1**, showed potent anti-proliferative activity against several cancer cell lines, but little with respect to tumor-promoting and proinflammatory activities. In contrast, **3** exhibited weaker growth inhibitory activity, and promoted inflammation and tumorigenesis. The binding affinity of **3** for PKCδ, which is involved in growth inhibition and apoptosis, was several times lower than those of **1** and **2**, possibly due to the absence of the hydrogen bond and CH/π interaction between its side chain and either Met-239 or Pro-241 in the PKCδ-C1B domain. These results suggest that both the aromatic ring and phenolic hydroxyl group can suppress the proinflammatory and tumor-promoting activities of **1** and, therefore, at least the aromatic ring in the side chain of **1** is indispensable for developing anti-cancer leads with potent anti-proliferative activity and limited side effects. In accordance with the binding affinity, the concentration of **3** necessary to induce PKCδ-GFP translocation to the plasma membrane and perinuclear regions in HEK293 cells was higher than that of **1** and **2**. However, the translocation profiles for PKCδ-GFP due to induction by **1**–**3** were similar.

## 1. Introduction

Naturally-occurring tumor-promoters such as phorbol esters, ingenol esters, teleocidins, and aplysiatoxins, exhibit potent tumor-promoting and proinflammatory activities [1], but also show anti-proliferative and proapoptotic activities towards several cancer cell lines [2,3]. The main targets of these compounds are protein kinase C (PKC) isozymes, a family of serine/threonine kinases involved in cellular signal transductions related to proliferation, differentiation, and apoptosis [4,5]. Tumor promoters bind to C1 domains (C1A and C1B) of conventional PKC (α, βI, βII, γ) and novel PKC (δ, Σ, η, θ) isozymes, and recruit them from the cytoplasm to the cell membrane to activate the enzymes [6]. Although PKC activation has been considered a potential strategy for cancer therapy [7], the application of PKC ligands to clinical uses is strictly limited by their severe adverse effects, such as tumor-promoting and proinflammatory activities.

By contrast, there are unique PKC activators with little tumor-promoting activity. The representative is bryostatin 1 (bryo-1), which was isolated from the marine bryozoan *Bugula neritina* [8]. Bryo-1 has been investigated in phase I and II clinical trials for the treatment of solid tumors, leukemia, and lymphoma, though most of them gave disappointing results [9,10]. 12-deoxyphorbol 13-phenylacetate (DPP) is also a non-tumor-promoting PKC ligand that inhibits the tumorigenesis induced by 12-*O*-tetradecanoylphorbol 13-acetate (TPA) in mouse skin [11]. Ingenol 3-angelate isolated from *Euphorbia peplus* was approved by the U.S. Food and Drug Administration (FDA) for topical treatment of precancerous actinic keratosis [12]. Although further studies on their mode of action and structural optimization are required, these have been hampered by low availability from natural sources and synthetic complexity. To overcome the short supply, several groups developed practical synthetic methods for bryo-1 and its congers [13,14,15]. Wender and colleagues reported a simplified analog of bryo-1 with stronger anti-proliferative activity than bryo-1 [16,17]. More recently, Baran’s group accomplished quite a short step synthesis of ingenol and phorbol [18,19].

**Figure 1:**
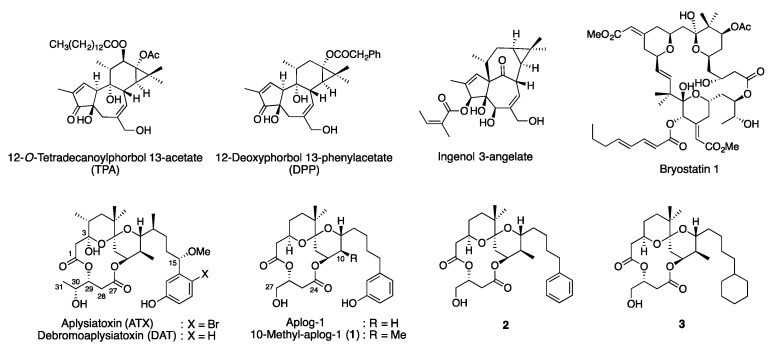
Structure of 12-*O*-tetradecanoylphorbol 13-acetate, 12-deoxyphorbol 13-phenylacetate, Ingenol 3-angelate, bryostatin 1, aplysiatoxin, debromoaplysiatoxin, and their simplified analogs.

As another way to circumvent the supply problem, we developed “aplog-1”, a simplified analog of tumor-promoting aplysiatoxin (ATX) [20], as a new anti-cancer seed (Figure 1) [21]. Aplog-1 showed anti-proliferative activity against several cancer cell lines, but, unlike ATX, did not exhibit tumor-promoting and proinflammatory activities [21]. Our comprehensive structure–activity studies revealed that methyl groups around the spiroketal moiety are important for inhibiting the growth of cancer cell lines [22,23,24]. In particular, the introduction of a chiral methyl group at position 10 of aplog-1 significantly enhances its anti-proliferative activity and affinity for the C1 domains of PKCs, while this derivative (**1**) barely promotes tumor formation in mouse skin or inflammation of mouse ears [23]. We also systematically investigated substituent effects on the phenolic ring of aplog-1, but we found that introducing substituents did not drastically influence aplog-1’s anti-proliferative and tumor-promoting activities [25]. Therefore, the role of the phenolic moiety itself on the biological activities of aplog-1 still remains unclear. This led us to synthesize new derivatives (**2**, **3**) to further examine the structure–activity relationship of the side chain of **1**. In this paper, we describe the effects of the aromatic ring and hydroxyl group found on the phenolic side chain of **1** on the various biological activities of **1**. We also determined the translocation profile of **1**–**3** for PKCδ, one of the PKC isozymes responsible for growth inhibition and apoptosis.

## 2. Results and Discussions

### 2.1. Anti-Proliferative Activity of ***2*** and ***3*** towards 39 Human Cancer Cell Lines

**Scheme 1:**
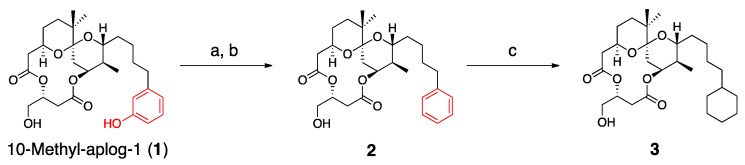
(**a**) *N*-phenyl-bis(trifluoromethanesulfonimide), triethylamine, CH_2_Cl_2_; (**b**) H_2_, 10% Pd/C, *N*,*N*-diisopropylethylamine, EtOH (65% in two steps); and (**c**) H_2_, 5% Rh/C, *i*PrOH (98%).

Compounds **2** and **3** were synthesized from **1** as described in Scheme 1. Selective triflation of the phenolic hydroxyl group followed by hydrogenation produced **2** (65% yield in two steps). Hydrogenation of the aromatic ring of **2** under the conditions reported by Sajiki’s group [26] gave **3** with high yield (98%). Since NOE pattern and ^1^H-NMR coupling constants of **2** and **3** were similar to those of **1** (Supplementary Materials), these structural modifications did not affect the configuration or the conformation of the spiroketal moiety and the macrolactone ring.

The anti-proliferative activities of **2** and **3** were evaluated using a panel of 39 human cancer cell lines established by Yamori and colleagues [27]. Growth inhibitory activity was expressed as a 50% growth inhibition (GI_50_), the concentration required to inhibit cell growth by 50% compared to an untreated control. Since ATX analogs exhibited cell line–specific anti-proliferative activity [24,28], GI_50_ values for seven cancer cell lines sensitive to them are listed in Table 1 (the rest of the data are provided in the Supplementary Materials). The GI_50_ values of **2** measured against some of these cell lines were lower than those of **1**, but we found the anti-proliferative activity of **2** to be equal to that of **1**. This was in agreement with a previous structure–activity study of aplog-1 [29]. On the other hand, **3** showed weaker growth inhibitory activity than **1**, except towards MKN-45. These results suggest that the aromatic ring is involved in inhibiting the growth of aplog-sensitive cancer cell lines.

### 2.2. Proinflammatory and Tumor-Promoting Activity of ***2*** and ***3***

**Figure 2:**
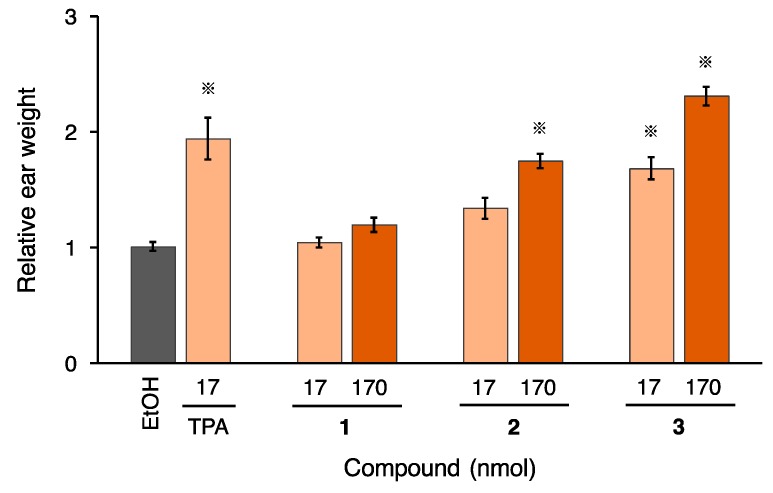
Change in relative weight of the mouse ear 24 h after the application of TPA, **1**, **2**, or **3**. Error bars show the standard error of four samples. ^※ ^*p* < 0.01 vs. vehicle (EtOH) group (Dunnett’s test).

Since the tumor-promoting and proinflammatory activities of phorbol esters are sensitive to the structure and hydrophobicity of their ester side chains [30,31], **2** and **3** may exhibit adverse effects, unlike **1**. Initially, we evaluated the proinflammatory activities of **2** and **3** in the mouse ear, because tumor promotion is related to chronic inflammation [32]. The ear of each ICR mouse was treated with each compound for 24 h. Proinflammatory activity was measured as an increase in the relative weight of each ear following treatment with each compound (Figure 2). In contrast to tumor-promoting TPA, **1** did not show significant proinflammatory activity even at a dose of 170 nmol. On the other hand, **2** induced inflammation at this concentration, although the inflammation was weak. Moreover, **3** exhibited marked proinflammatory activity even at a dose of only 17 nmol like TPA.

**Figure 3:**
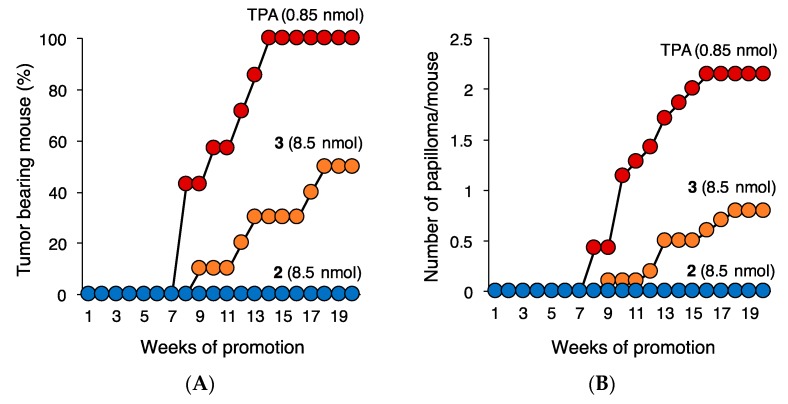
Tumor-promoting activity of TPA (0.85 nmol), **2** (8.5 nmol), and **3** (8.5 nmol). The back of each female six-week-old ICR mice was shaved with surgical clippers. From a week after the initiation by a single application of 780 nmol of DMBA in 0.1 mL acetone, each compound (0.85 or 8.5 nmol) in 0.1 mL acetone was applied twice a week from weeks 1–20. The TPA group consisted of seven mice, and other groups consisted of ten mice. (**A**) Tumor-bearing mice (%); (**B**) Number of papilloma per mouse.

The tumor-promoting activities of **2** and **3** were investigated in a two-stage carcinogenesis test on mouse skin (Figure 3). We have already confirmed that **1** was negative for papilloma formation even in the presence of a five-fold excess of TPA in another experiment [23]. The skin on the back of ICR mice was treated with a single dose of 780 nmol of 7,12-dimethylbenz[*a*]anthracene (DMBA) and, one week later, with 8.5 nmol of **2** or **3** (10-fold excess of TPA) twice a week. In the positive control experiment using TPA (0.85 nmol), the first papilloma appeared in week 8, and all mice bore papillomas by week 14. The application of 8.5 nmol **2** did not induce any papillomas. On the other hand, 8.5 nmol of **3** significantly enhanced papilloma formation, and the percentage of tumor-bearing mice reached 50% by week 18. These results suggest that both the phenolic hydroxyl group and aromatic ring in **1** suppressed the proinflammatory and tumor-promoting activities.

### 2.3. Binding Affinity of ***2*** and ***3*** for PKCδ-C1B Domain

PKCδ is related to growth inhibition and apoptosis [33,34,35], and is involved in the anti-cancer and anti-tumor-promoting activities of both bryo-1 and ingenol 3-angelate [36,37]. Moreover, our previous study showed that the ability to bind to PKCδ is closely correlated to anti-proliferative activity against aplog-sensitive cancer cell lines [38]. Thus, we measured the affinity of **2** and **3** for PKCδ using a competitive binding assay with [^3^H]phorbol 12,13-dibutyrate (PDBu) as described by Sharkey and Blumberg [39]. Since whole PKCδ protein produced by molecular biology techniques were occasionally unfolded and unstable, we adopted a more reliable assay using our synthetic PKCδ-C1B peptide [40]. Although PKCδ has both C1A and C1B domains, the main binding site for PKC ligands has been found to be the C1B domain [40,41,42]. The binding affinity of several PKC ligands for δ-C1B peptide is almost equal to that for full-length PKCδ [23,40,43]. As shown in Table 2, the affinity of **2** for δ-C1B was rather weak, but comparable to that of **1**. By contrast, **3** showed a binding affinity for δ-C1B lower by one order of magnitude, which could account for its weak anti-proliferative activity shown in Table 1.

**Figure 4:**
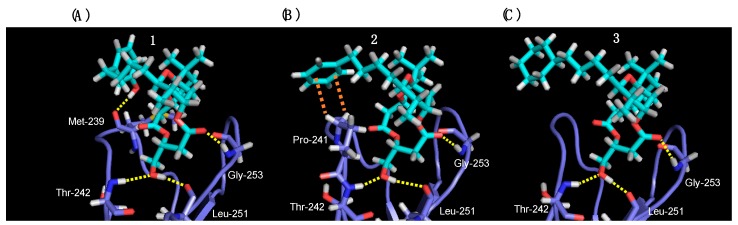
Structures of the PKCδ-C1B domain in complex with **1** (**A**), **2** (**B**), and **3** (**C**). Each compound is drawn as a stick model. δ-C1B is depicted in cartoon form, and amino acid residues that could form hydrogen bonds or be involved in CH/π interactions with the aromatic ring of each compound are represented in stick model colored purple (carbon), red (oxygen), blue (nitrogen), yellow (surfer), and white (hydrogen). Yellow and orange dashed lines represent hydrogen bonds and CH/π interactions, respectively.

To predict the binding mode between each derivative and δ-C1B, we performed docking simulation followed by refinement using molecular dynamics (MD) as reported previously [44]. Representative complex structures were shown in Figure 4. Based on previous structure-activity studies of ATX, the hydrophilic moiety at positions 1 and 27–31 including two ester groups and a hydroxyl group was thought to play key roles in the binding to PKC C1 domains [45,46,47]. In our docking models, 24-C=O and 27-OH groups of **1**–**3** could form three hydrogen bonds with Thr-242, Leu-251, and Gly-253 on δ-C1B. In addition, the phenolic hydroxyl group in the side chain of **1** formed another hydrogen bond with the C=O group of Met-239. These results are consistent with a model for binding between ATX and δ-C1B [44]. Instead of this hydrogen bond, the aromatic ring of **2** could be involved in the CH/π interaction with Pro-241, whereas there was no significant interaction between the cyclohexyl moiety of **3** and δ-C1B. These models could well explain the differences among **1**–**3** in the binding affinity for δ-C1B.

### 2.4. Structure–Activity Relationship in the Phenolic Moiety of 10-Methyl-aplog-1 (***1***)

The phenolic hydroxyl group and aromatic ring in the side chain of **1** maintained its affinity for PKCδ through a hydrogen bond or CH/π interaction with Met-239 or Pro-241 of δ-C1B, resulting in potent anti-proliferative activity against several cancer cell lines. Moreover, this moiety suppressed adverse effects, such as proinflammatory and tumor-promoting activities. The increase in hydrophobicity of the ester side chains of phorbol esters is considered to enhance their tumor-promoting activity [30,31], and tumor-promoting **3** is more hydrophobic than non-tumor-promoting **1** and **2** (calculated log *P* of **1**, 3.4; of **2**, 4.1; of **3**, 5.3). However, tumor-promoting and proinflammatory activities of ATX analogs did not merely depend on the hydrophobicity, because introduction of a bromine or iodine atom onto the aromatic ring of aplog-1 or **1** did not promote these activities [24,25]. Thus, the interaction between aplog’s side chain and δ-C1B could be involved in suppression of proinflammatory- and tumor-promoting activities. On the other hand, ATX showed tumor-promoting activity despite the presence of the phenolic group in its side chain. Our recent studies suggested that the hydroxyl group at position 3 and the methoxy group at position 15 of ATX could enhance tumor-promoting activity [23,48]. These groups might inhibit the interaction of ATX’s side chain and δ-C1B by inducing conformational change or steric hindrance.

### 2.5. Translocation of PKCδ-GFP Induced by ***1***–***3***

As mentioned above, the activation of PKCs by PKC ligands is triggered by translocation from the cytoplasm to the membrane fraction. The differences among PKC ligands in their biological activities are attributed to their effect on the translocation profile of PKCδ [31,49]. Blumberg and colleagues reported that bryo-1 and DPP with anti-tumor-promoting activity induced the translocation of green fluorescent protein-tagged PKCδ-PKCδ-GFP) to the nuclear membrane in Chinese hamster ovary (CHO) cells, whereas tumor promoters, such as TPA, caused its recruitment predominantly to the plasma membrane [49]. In human embryonic kidney 293 (HEK293) cells, bryo-1 also caused PKCδ-GFP to localize to the nuclear membrane and endoplasmic reticulum [50]. On the other hand, TPA caused PKCδ-GFP recruitment predominantly to the plasma membrane and the nuclear envelope [50,51], whereas PDBu, a less hydrophobic TPA analog, promoted its accumulation in the Golgi apparatus [51]. Since the hydrophobicity of the side chain of phorbol esters markedly affected PKCδ localization [31,49], we speculated that tumor-promoting **3** may show a translocation profile different from that of the less hydrophobic, non–tumor-promoting **1** and **2**.

**Figure 5:**
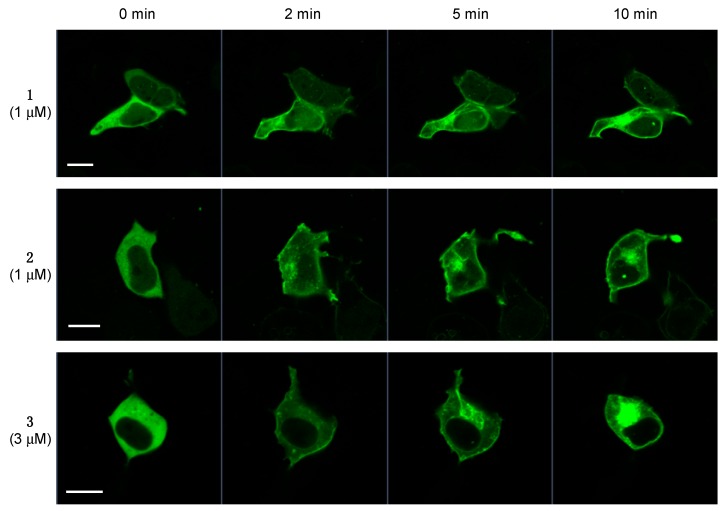
Translocation of PKCδ-GFP in HEK293 cells induced by **1** (1 μM), **2** (1 μM), and **3** (3 μM). Fluorescence images of HEK293 cells expressing PKCδ-GFP 0, 2, 5, and 10 min after treatment with each compound are shown. Scale bar: 10 μm.

The translocation assay in living HEK293 cells transfected with PKCδ-GFP was shown in Figure 5. Stimulation by 1 μM of either **1** or **2** induced the translocation of PKCδ-GFP to the plasma membrane and the perinuclear region, possibly to the Golgi apparatus. However, stimulation by 1 μM **3** barely induced translocation of PKCδ-GFP (data not shown), reflecting low affinity for PKCδ, but a higher concentration (3 μM) of **3**, was sufficient to recruit it to the plasma membrane and near the nucleus, in a manner similar to **1** and **2**. Interestingly, their translocation profile resembled that of tumor-promoting phorbol esters rather than bryo-1. These results indicate that the localization pattern of PKCδ is not related to the tumor-promoting activity of ATX analogs.

Recently, Kazanietz’s group demonstrated that bryo-1 prevents TPA-induced apoptosis by causing PKCδ to localize to the nuclear membrane, but that bryo-1 itself did not trigger any biological responses [52]. Keck and Blumberg also reported that bryo-1 itself exhibited only weak anti-proliferative activity, but antagonized the anti-proliferative activity of TPA [53]. In addition, most cancer clinical trials using bryo-1 have yielded disappointing results [9,10]. Therefore, tumor promoter-like PKC ligands that recruit PKCδ to the plasma membrane or the Golgi apparatus may be suitable as anti-cancer drugs. Indeed, PKCδ activation in either the plasma membrane or Golgi apparatus induces apoptosis [52,54]. Compound **1** is a novel PKC ligand that activates PKCδ in a manner similar to tumor promoters despite the absence of tumor-promoting and proinflammatory activities. Further investigations to rationalize the pleiotropic effects of PKC ligands are underway in our laboratory.

## 3. Materials and Methods

### 3.1. General Remarks

The following spectroscopic and analytical instruments were used: digital polarimeter, DIP-1000 (Jasco, Tokyo, Japan); ^1^H- and ^13^C-NMR, Avance III 500 (reference TMS, Brucker, Rheinstetten, Germany); HPLC, model 600E with a model 2487 UV detector (Waters, Milford, MA, USA); HR-ESI-qTOF-MS, Waters Xevo G2-S qTOF (Waters, Milford, MA, USA); and confocal laser scanning fluorescence microscopy, LSM700 (Carl Zeiss, Jena, Germany). HPLC was carried out on YMC-Pack SIL SL12S05-2510WT (10 mm i.d. × 250 mm, Yamamura Chemical Laboratory, Kyoto, Japan). Wakogel C-200 (silica gel, Wako Pure Chemical Laboratory, Osaka, Japan) was used for column chromatography. [^3^H]PDBu (18.7 Ci/mmol) was custom synthesized by Perkin-Elmer Life Science Research Products (Boston, MA, USA). PKCδ-C1B peptide was synthesized as reported previously [41]. All other chemicals and regents were purchased from chemical companies and used without further purification. The purity of **2** and **3** was greater than 95% as determined by ^1^H- and ^13^C-NMR (Supplementary Materials). All animal use procedures were approved by Kyoto University Animal Experimentation Committee and performed according to its guidelines.

### 3.2. Synthesis of ***2*** and ***3***

Compound **2**. Triethyl amine (5.5 μL, 23.8 μmol, 1.2 equiv.) and *N*-phenyl-bis(trifluoromethane-sulfonimide) (8.5 mg, 23.8 μmol, 1.2 equiv.) were added to a solution of 10-methyl-aplog-1 (**1**) (10 mg, 19.8 μmol) in CH_2_Cl_2_ (150 μL) at room temperature. The reaction mixture was stirred at room temperature for 8.5 h and poured into 1 mL water. The aqueous layer was extracted four times with 2 mL EtOAc. The combined organic layers were washed with brine, dried over Na_2_SO_4_, filtered, and concentrated in vacuo. The residue was purified by column chromatography (silica gel, 25% EtOAc/hexane) to produce a crude mixture that consists mostly of the desired trifulate (10.5 mg). We added 25 μL *N*,*N*-diisopropylethylamine to a mixture of the trifulate (10.5 mg) and 10% Pd/C (7.9 mg) (wet support, Degussa type E101 NE/W, Sigma-Aldrich, St. Louis, MO, USA) in 300 μL EtOH and stirred vigorously under a H_2_ atmosphere at room temperature for 4 h. The mixture was filtered, and the filtrate was concentrated in vacuo. The residue was purified by column chromatography (silica gel, 25% EtOAc/hexane), and further purified by HPLC (column, YMC-Pack SIL SL-12S05-2510WT; solvent, *i*PrOH/CHCl_3_/hexane = 2:18:80; flow rate, 3 mL/min; pressure, 660 psi; UV detector, 254 nm; retention time, 18 min) to produce **2** (6.3 mg, 12.9 μmol, 65% in two steps) as a clear oil. ^1^H-NMR (500 MHz, CDCl_3_, 0.0049 M; ppm) δ 0.79 (3H, d, *J* = 6.9 Hz), 0.86 (3H, s), 0.96 (3H, s), 1.29–1.38 (2H, m), 1.39–1.62 (8H, m), 1.70 (1H, m), 1.71 (1H, dd, *J* = 15.5, 4.0 Hz), 2.24 (1H, m), 2.39 (1H, dd, *J* = 13.0, 10.9 Hz), 2.49–2.55 (2H, m), 2.62 (2H, t, *J* = 7.7 Hz), 2.73 (1H, dd, *J* = 16.8, 3.4 Hz), 2.81 (1H, dd, 16.8, 11.2 Hz), 3.72 (1H, m), 3.79 (1H, ddd, *J* = 11.9, 5.9, 3.8 Hz), 3.87 (1H, tt, *J* = 10.9, 2.9 Hz), 3.94 (1H, m), 5.01 (1H, m), 5.18 (1H, m), 7.15 (1H, t, *J* = 1.3 Hz), 7.16–7.22 (2H, m), 7.26–7.28 (2H, m); ^13^C-NMR (125 MHz, CDCl_3_, 0.0049 M; ppm) δ 13.2, 21.4, 24.2, 26.0, 26.5, 27.3, 31.2, 32.3, 34.6, 36.0, 36.8, 36.9, 36.9, 42.8, 64.5, 68.3, 70.5, 71.8, 73.0, 99.9, 125.5, 128.2 (2C), 128.5 (2C), 143.1, 169.8, 171.5; IR (KBr; cm^−1^) 3447, 2360, 1726, 1294, 1273, 1198, 1060; HR-ESI-qTOF-MS: *m/z* 487.2698 [M − H]^−^ (calculated for C_28_H_39_O_7_, 487.2696); ${\left[\alpha \right]}_{D}^{21.7}$ +66.3° (*c* 0.13, CHCl_3_).

Compound **3**. The mixture of **2** (7.1 mg, 14.5 μmol) and 5% Rh/C (4.6 mg) (wetted with 55% water, Tokyo Chemical Industry) in *i*PrOH (350 μL) was vigorously stirred under a H_2_ atmosphere at room temperature for 17 h. The mixture was filtered and the filtrate was concentrated in vacuo. The residue was purified by column chromatography (silica gel, 25% EtOAc/hexane) to afford **3** (7.0 mg, 14.2 μmol, 98%) as clear oil. ^1^H-NMR (500 MHz, CDCl_3_, 0.0057 M; ppm) δ 0.79 (3H, d, *J* = 6.9 Hz), 0.83–0.92 (5H, m), 1.00 (3H, m), 1.12–1.57 (17H, m), 1.59–1.73 (6H, m), 2.25 (1H, t, *J* = 5.9 Hz), 2.37 (1H, dd, *J* = 13.0, 10.9 Hz), 2.49–2.54 (2H, m), 2.72 (1H, dd, *J* = 16.8, 3.5 Hz), 2.80 (1H, dd, *J* = 16.8, 11.2 Hz), 3.71 (1H, m), 3.79 (1H, ddd, *J* = 12.0, 5.9, 3.9 Hz), 3.84–3.93 (2H, m), 5.01 (1H, m), 5.17 (1H, m); ^13^C-NMR (125 MHz, CDCl_3_, 0.0057 M; ppm) δ 13.2, 21.4, 24.7, 26.0, 26.5 (3C), 26.6, 26.9, 27.3, 32.6, 33.5, 33.5, 34.6, 36.8, 36.9, 36.9, 37.6, 37.6, 42.7, 64.5, 68.4, 70.5, 71.8, 73.0, 99.8, 169.8, 171.5; IR (KBr; cm^−1^) 3470, 2923, 2852, 1728, 1295, 1273, 1199, 1074, 1061; HR-ESI-qTOF-MS: *m/z* 493.3170 [M − H]^−^ (calculated for C_28_H_45_O_7_, 493.3165); ${\left[\alpha \right]}_{D}^{21.7}$ + 73.8° (*c* 0.13, CHCl_3_).

### 3.3. Measurement of Cell Growth Inhibition

We employed a panel of 39 human cancer cell lines established by Yamori and colleagues according to the National Cancer Institute (NCI) method with modifications [27]. We measured the inhibitory activity of test compounds towards cell growth as reported previously [55]. In brief, the cells were seeded on 96-well plates in Roswell Park Memorial Institute (RPMI) 1640 medium supplemented with 5% fetal bovine serum and allowed to attach overnight. The cells were incubated with each test compound for 48 h. Cell growth was estimated using the sulforhodamine B assay. The 50% growth inhibition (GI_50_) parameter was calculated as reported previously [56]. Absorbance was measured for the control well (*C*) and the test well (*T*) at 525 nm along with that for the test well at time 0 (*T*_0_). Cell growth inhibition (% growth) by each concentration of drug (10^−8^, 10^−7^, 10^−6^, 10^−5^, and 10^−4^) was calculated as 100[(*T* − *T*_0_)/(*C* − *T*_0_)] using the average of duplicate points. By processing these values, we determined each GI_50_ value, defined as 100[(*T* − *T*_0_)/(*C* − *T*_0_)] = 50.

### 3.4. Mouse Ear Swelling Test

We applied either a solution of each test compound in EtOH (10 μL) or EtOH as a control to the right ear of five-week-old female ICR mice (Shimizu Laboratory Supplies, Kyoto, Japan) using a micropipette. A volume of 5 μL was delivered to both the inner and outer surfaces of the ear. After 24 h, a disk (0.8 cm square) was obtained from the ear and weighed. The proinflammatory activity of each compound was determined by measuring relative ear weight, i.e., the weight of a right ear disk relative to the weight of a left ear disk. Each group consisted of four mice.

### 3.5. Two-Stage Carcinogenesis Experiment

The back of each six-week-old female ICR mice (Shimizu Laboratory Supplies) was shaved with surgical clippers one day before DMBA treatment. From a week after initiation by a single dose of 780 nmol DMBA in 0.1 mL acetone, we administered 0.85 nmol of TPA in 0.1 mL acetone, 8.5 nmol of **2** in 0.1 mL acetone, or 8.5 nmol of **3** in 0.1 mL acetone to each mouse twice a week from week 1 to 20. The number of skin papilloma >1 mm in diameter was counted every week. Each group consisted of 7–10 mice.

### 3.6. Inhibition of the Specific Binding of [^3^H]PDBu to PKCδ-C1B Peptide

The binding of [^3^H]PDBu to PKCδ-C1B peptide was evaluated by the procedure of Sharkey and Blumberg with modifications [43] using 50 mM Tris-maleate buffer (pH 7.4), 13.8 nM PKCδ-C1B peptide, 20 nM [^3^H]PDBu (18.7 Ci/mmol), 50 μg/mL 1,2-dioleoyl-*sn*-glycero-3-phospho-l-serine sodium salt (Sigma-Aldrich), 3 mg/mL bovine γ-globulin (Sigma-Aldrich), and various concentrations of an inhibitor. Binding affinity was determined based on the concentration required to cause 50% inhibition of the specific binding of [^3^H]PDBu, the IC_50_, which was calculated by log-probit regression analysis. The inhibition constant *K*_i_ was calculated by the method of Sharkey and Blumberg [41].

### 3.7. Molecular Docking Simulation

Molecular dynamics (MD) simulations for the POPS–ligand–PKCδ-C1B ternary complex in water were carried out as described previously [44]. All MD simulations were performed using the GROMACS software package (version 5.1.4) [57]. After equilibrating and relaxing the system, we performed a 30 ns NPT (constant number of atoms, pressure, and temperature) simulation without any position restraint with a 2 fs time step.

### 3.8. Translocation of PKCδ-GFP

We transfected HEK293 cells with a plasmid encoding PKCδ-GFP using Lipofectamine^®^ 3000. The transfected cells were cultured for 20–48 h for maximal fluorescence. Translocation of GFP-tagged PKCδ was triggered by the addition of a test compound to the culture medium (final DMSO concentration 0.05%–0.15%) to obtain the appropriate final concentration. The fluorescence of GFP was monitored by confocal laser scanning fluorescence microscopy at 488 nm excitation with a 494-nm-long pass beam splitter.

## 4. Conclusions

New derivatives (**2**, **3**) of 10-methyl-aplog-1 (**1**) were prepared to examine in detail the role of the phenolic side chain in the various biological activities of this compound. The biological activities of **2** were similar to those of **1** except for its weak proinflammatory effects. On the other hand, **3** showed a slightly weaker growth inhibitory activity than that of **1**, and significantly induced inflammation and tumorigenesis. Moreover, the binding affinity of **3** for PKCδ-C1B peptide was lower than those of **1** and **2**, possibly due to the lack of the hydrogen bond and CH/π interaction between its side chain and Met-239 or Pro-241 residue of δ-C1B. Overall, at least the aromatic ring in the side chain of **1** is indispensable for developing anti-cancer leads with potent anti-proliferative activity and few side effects.

Hitherto, the tumor-promoting activity of PKC ligands has been attributed to the pattern of PKCδ localization of TPA, primarily plasma membrane translocation. However, all derivatives (**1**–**3**) recruited PKCδ-GFP to the plasma membrane and near the nucleus, similarly to tumor-promoting phorbol esters rather than bryo-1, which lacks tumor-promoting activity. Bryo-1 inhibited TPA-induced biological responses, such as tumorigenesis, growth inhibition, and apoptosis, but did not itself significantly show anti-proliferative or proapoptotic activities. Thus, we concluded that **1** could be a new TPA-like activator of PKCδ that lacks tumor-promoting and proinflammatory activities.

## Figures and Tables

**Table 1:** Growth inhibitory activity of **1**, **2**, and **3** towards aplog-sensitive cancer cell lines.

Cancer Type	Cell Line	GI_50_ (log M)		
		1 ^a^	2	3
Breast	HBC-4	−7.48	−7.76	−7.20
	MDA-MB-231	−6.90	−5.63	−5.68
Colon	HCC2998	−6.47	−6.21	−6.08
Lung	NCI-H460	−7.07	−7.09	−6.85
	A549	−6.01	−6.12	−5.78
Stomach	St-4	−6.24	−5.93	−5.89
	MKN45	−4.97	−6.51	−6.13
Average for these seven cell lines		−6.45	−6.46	−6.23

^a^ Cited from Ref. [23].

**Table 2:** *K*_i_ values for the inhibition of [^3^H]PDBu binding by **1**, **2**, and **3**.

*K*_i_ (nM) for PKCδ C1B		
**1** ^a^	**2**	**3**
0.46	0.87 (0.18) ^b^	3.8 (0.67) ^b^

^a^ Cited from [23]. ^b^ Standard deviation from four separate experiments.

## Supplementary Materials

The following data are available online. ^1^H and ^13^C-NMR spectra of **2** and **3**, and growth inhibitory activities of **2** and **3** towards 39 human cancer cell lines.
