# Supplementary material for: Loss of the Phenolic Hydroxyl Group and Aromaticity from the Side Chain of Anti-Proliferative 10-Methyl-aplog-1, a Simplified Analog of Aplysiatoxin, Enhances Its Tumor-Promoting and Proinflammatory Activities
Source: Molecules. 2017 Apr 13;22(4):631. doi: 10.3390/molecules22040631 (PMC6153940; doi:10.3390/molecules22040631)
Supplement: Supplementary file 1 [file molecules-22-00631-s001.pdf]

Supporting Information

Loss of the phenolic hydroxyl group and aromaticity from the side chain of anti-proliferative 10-methyl-aplog-1, a simplified analog of aplysiatoxin, enhances its tumor-promoting and proinflammatory activities

Yusuke Hanaki [1], Masayuki Kikumori [1], Harukuni Tokuda [1], Mutsumi Okamura [2], Shingo Dan [2], Naoko Adachi [3], Naoaki Saito [3], Ryo C. Yanagita [4], and Kazuhiro Irie [1,*]

[1] *Division of Food Science and Biotechnology, Graduate school of Agriculture, Kyoto University, Kyoto 606-8502, Japan*

[2] *Division of Molecular Pharmacology, Cancer Chemotherapy Center, Japanese Foundation for Cancer Research, Tokyo 135-8550, Japan*

[3] *Laboratory of Molecular Pharmacology, Biosignal Research Center, Kobe University, Kobe 657-8501, Japan*

[4] *Faculty of Agriculture, Depertment of Applied Biological Science, Kagawa University, Kagawa 761-0795, Japan*

Contents

I. ^{1}H and ^{13}C NMR spectra of **2** and **3**

II. NOESY spectra of **1–3**

III. MS spectra of **2** and **3**

IV. IR spectra of **2** and **3**

V. Growth inhibitory activities of **2** and **3** towards 39 human cancer cell lines

I. ^{1}H and ^{13}C NMR spectra of **2** and **3**

^{1}H-1D NMR spectrum of **2** (500 MHz, $CDCl_3$, 0.0049 M)

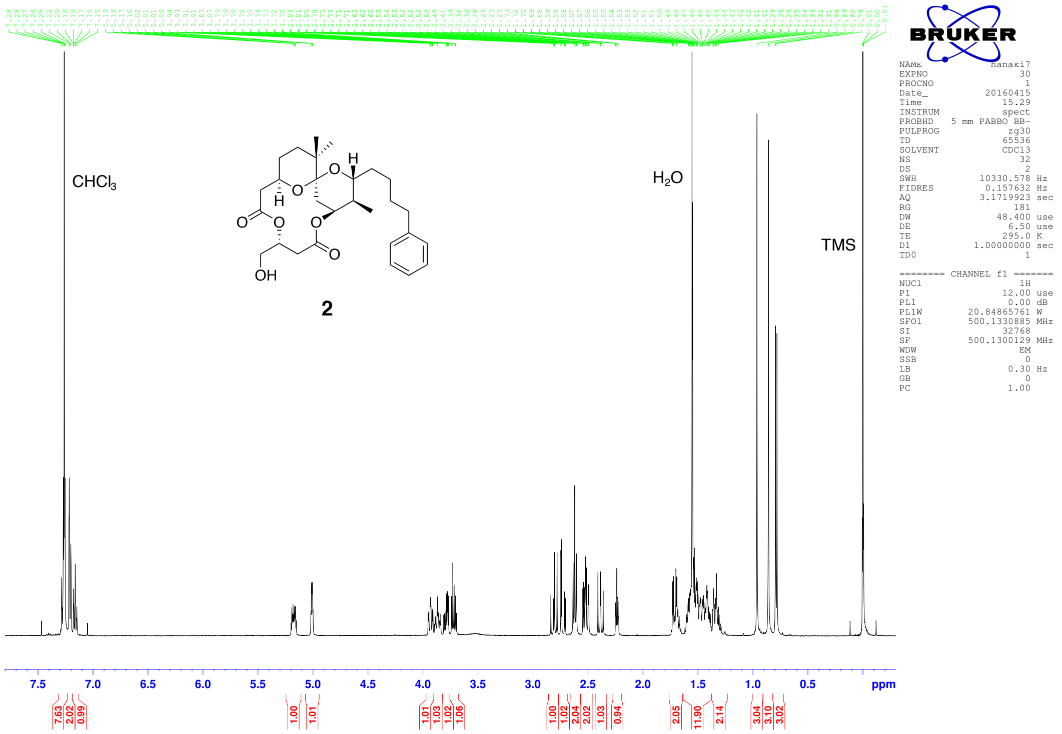

^{13}C-1D NMR spectrum of **2** (125 MHz, $CDCl_3$, 0.0049 M)

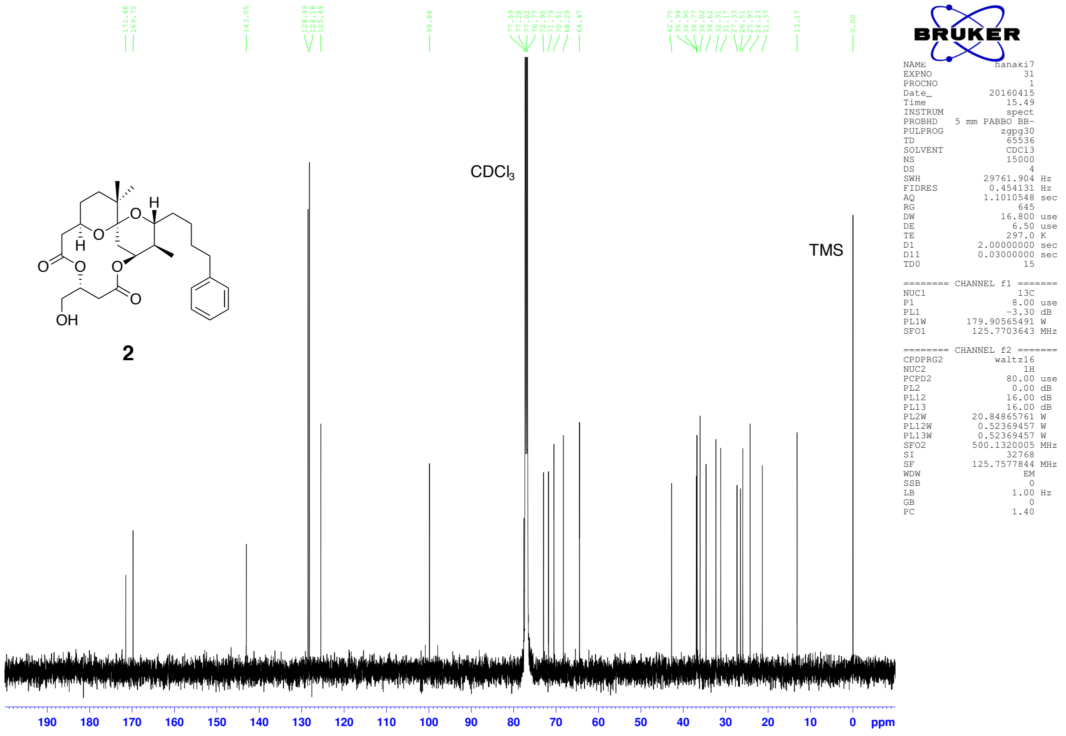

^{1}H-1D NMR spectrum of **3** (500 MHz, $CDCl_3$, 0.0057 M)

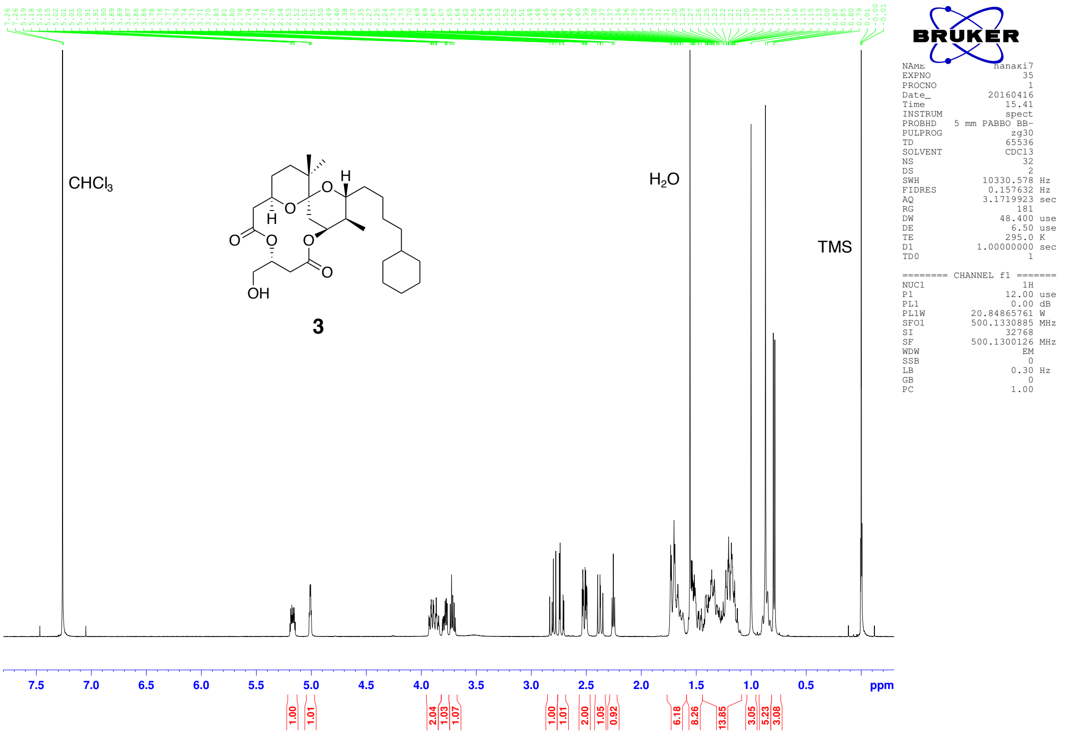

^{13}C-1D NMR spectrum of **3** (500 MHz, $CDCl_3$, 0.0057 M)

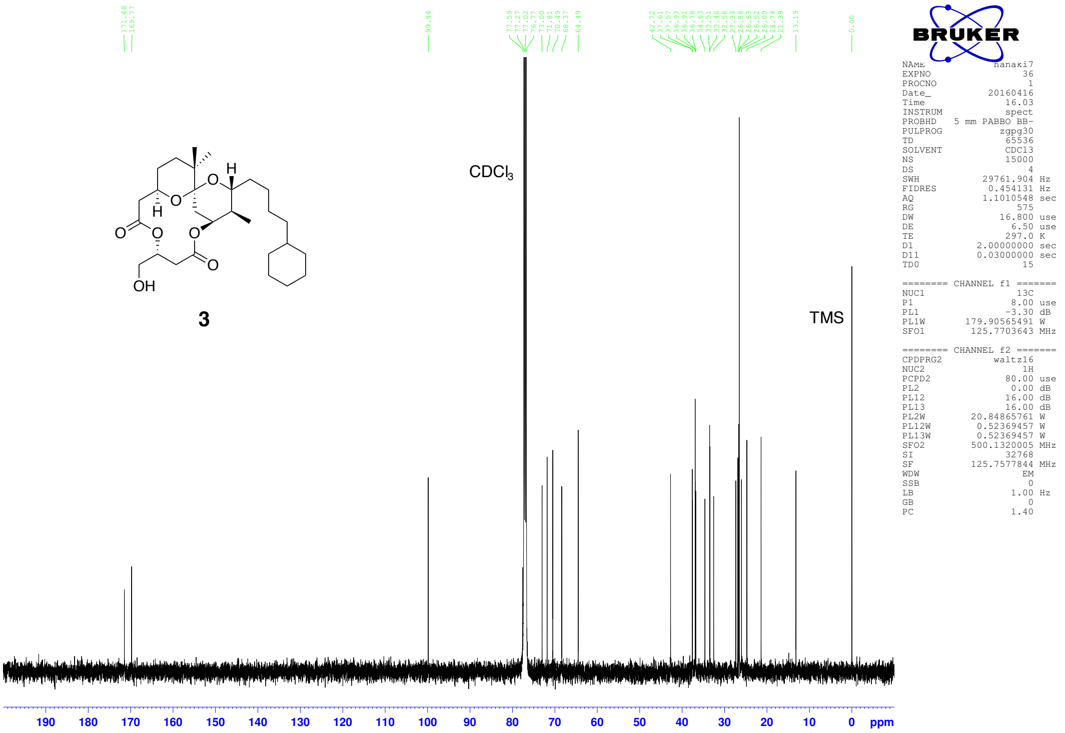

II. NOESY spectra of **1–3**

NOESY spectrum of **1** (500 MHz, $CDCl_3$, 0.010 M)

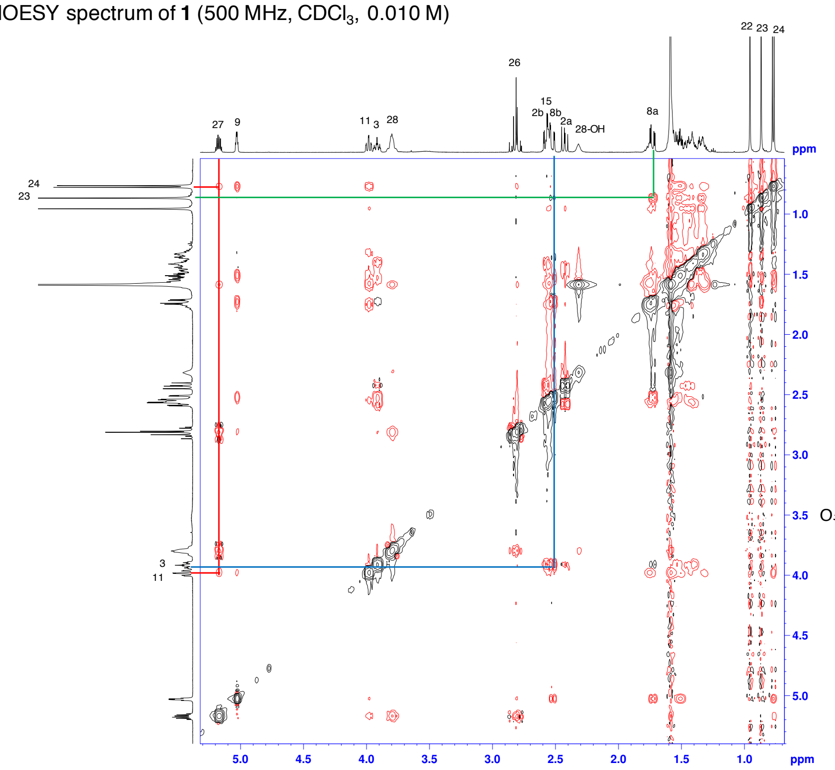

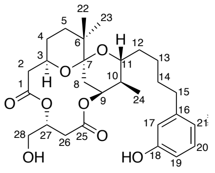

10-Methyl-aplog-1 (**1**)

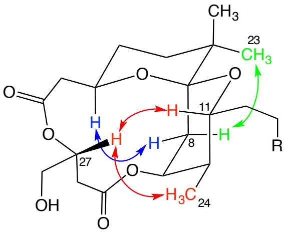

NOESY spectrum of **2** (500 MHz, $CDCl_3$, 0.0053 M)

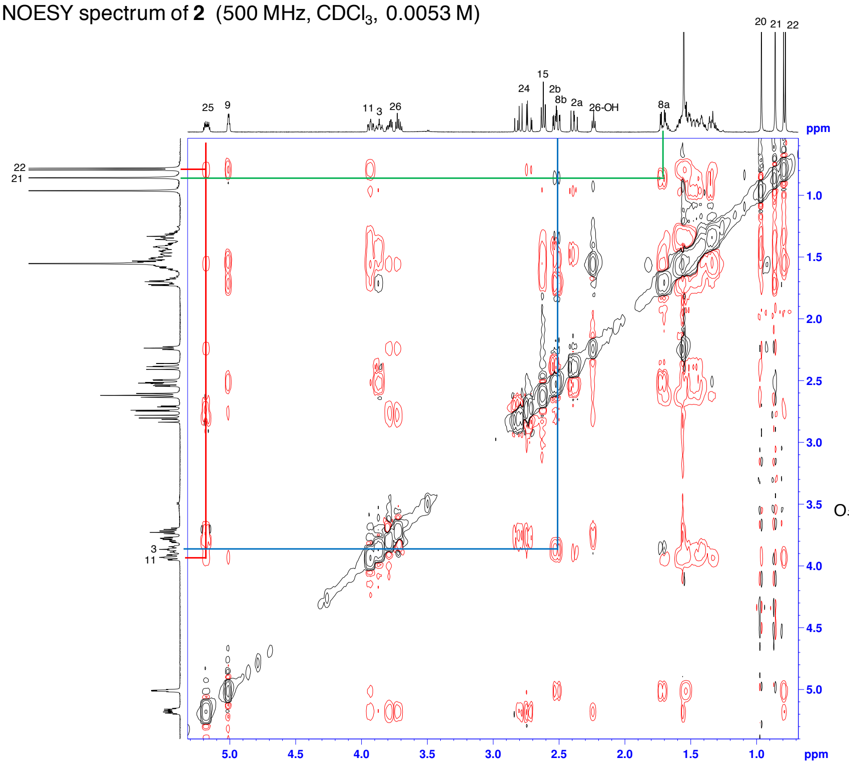

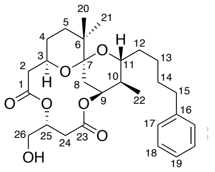

2

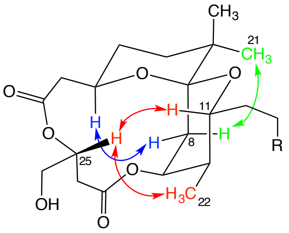

NOESY spectrum of **3** (500 MHz, $CDCl_3$, 0.0052 M)

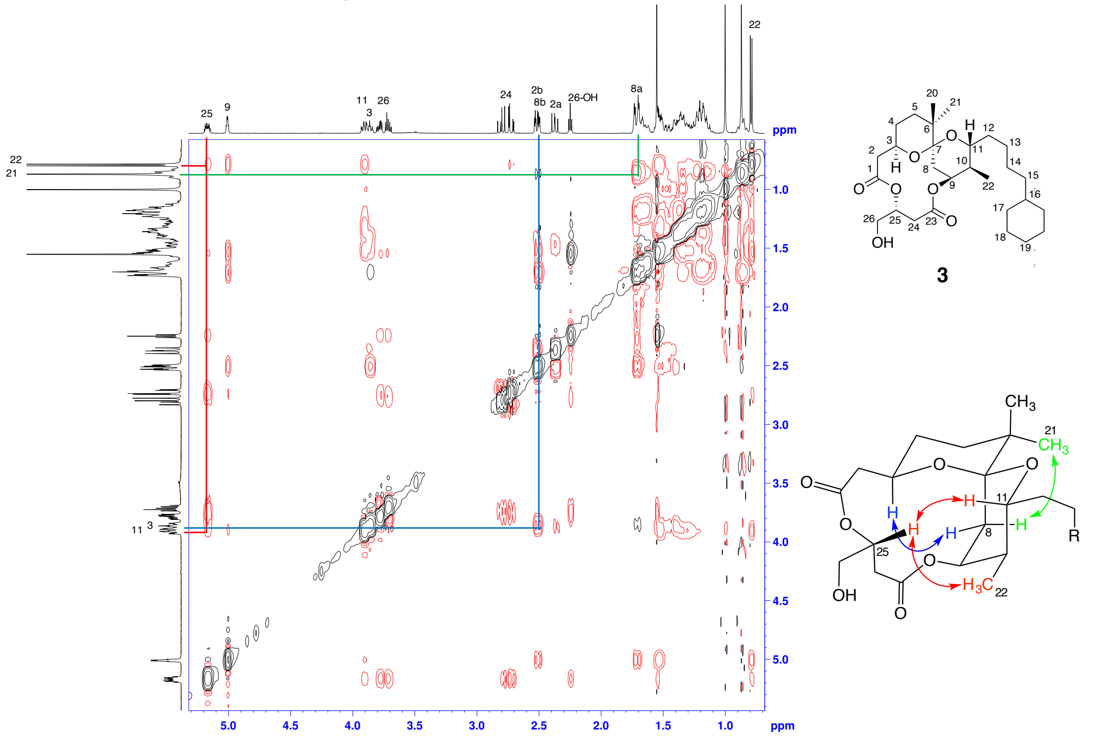

III. MS spectra of **2** and **3**

ESI-MS spectrum of **2**

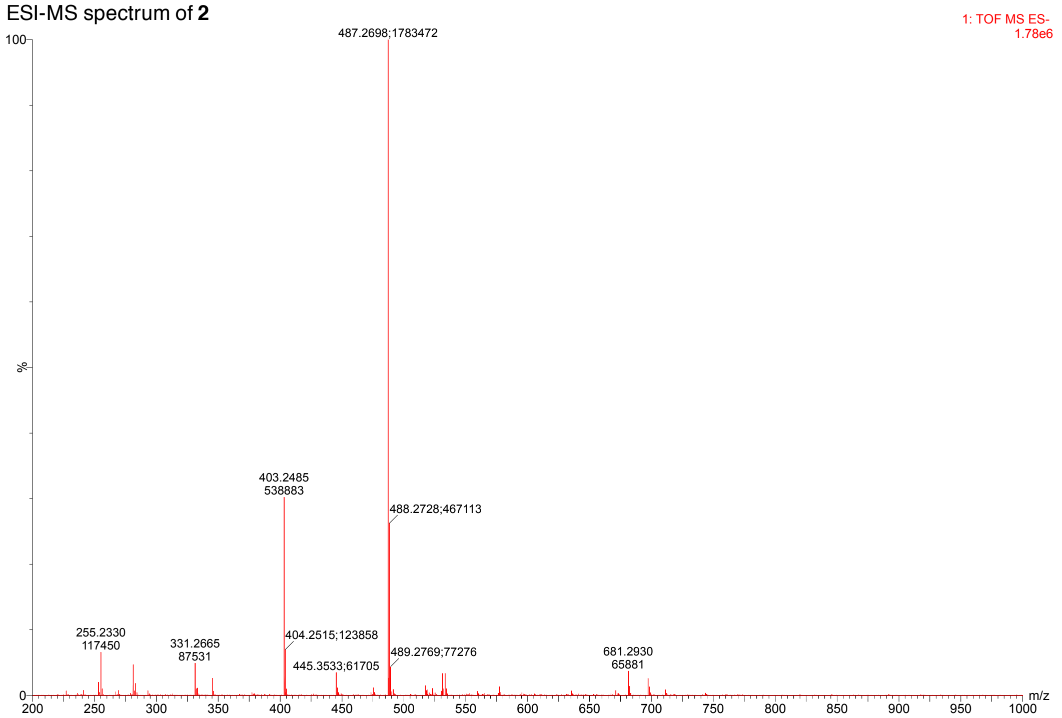

ESI-MS spectrum of **3**

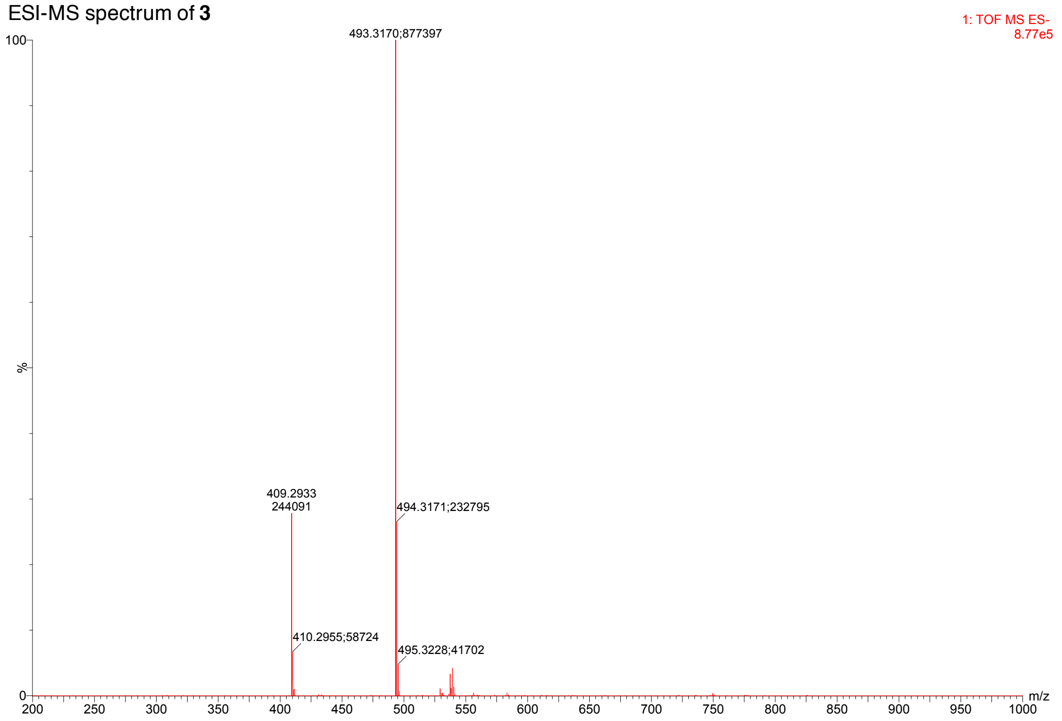

IV. IR spectra of **2** and **3**

IR spectrum of **2**

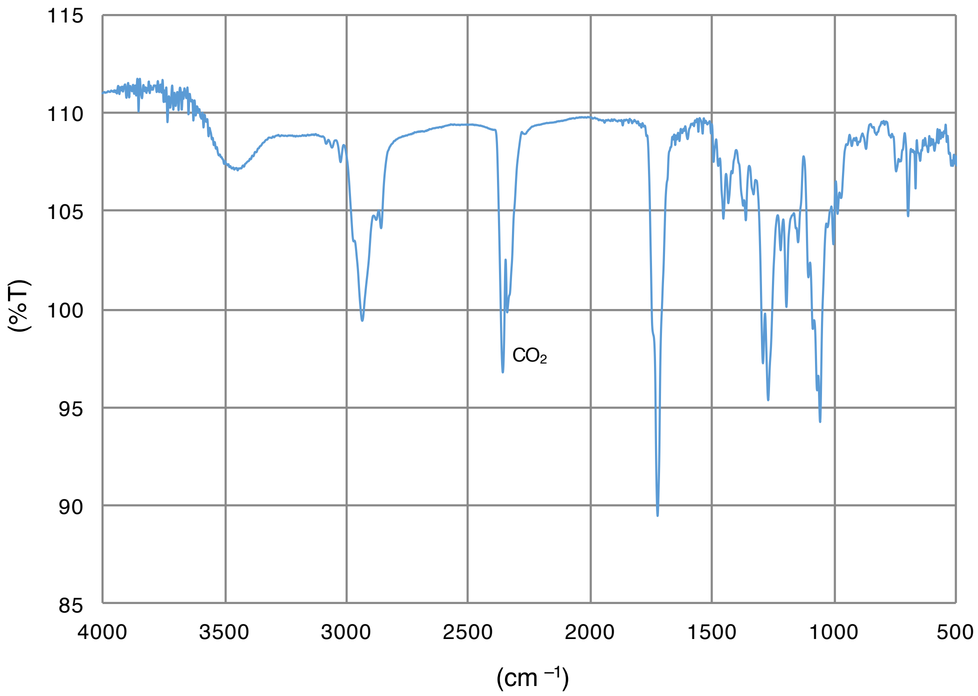

IR spectrum of **3**

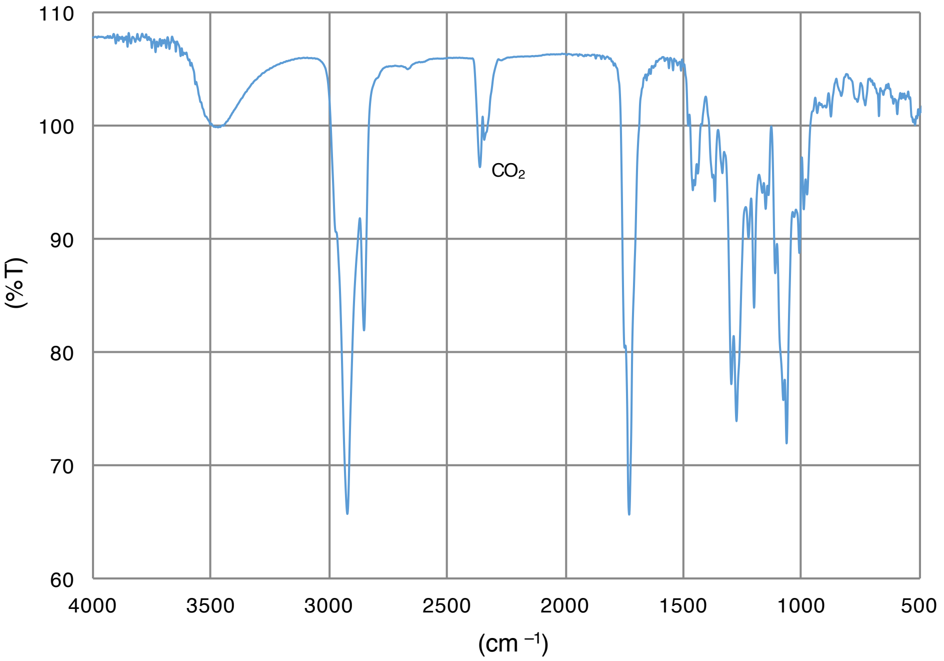

V. Growth inhibitory activities of **2** and **3** towards 39 human cancer cell lines

cancer cell lines		log GI_{50} (M)		
		**1**[a]	**2**	**3**
**Breast**	HBC-4	–7.48	–7.76	–7.20
	BSY-1	–4.91	–4.88	–4.99
	HBC-5	–4.82	–4.83	–4.90
	MCF-7	–4.84	–4.99	–5.29
	MDA-MB-231	–6.90	–5.63	–5.68
**CNS**	U251	–4.79	–4.76	–4.80
	SF-268	–4.83	–4.78	–4.86
	SF-295	–4.98	–4.90	–5.20
	SF-539	–4.88	–4.90	–4.91
	SNB-75	–4.92	–4.91	–4.89
	SNB-78	–6.05	–4.81	–4.79
**Colon**	HCC2998	–6.47	–6.21	–6.08
	KM-12	–4.82	-4.87	–4.99
	HT-29	–4.81	–4.95	–5.05
	HCT-15	–4.87	–4.78	–4.76
	HCT-116	–4.89	–5.02	–5.07
**Lung**	NCI-H23	–4.87	–4.75	–4.74
	NCI-H226	–6.15	–4.94	–5.02
	NCI-H522	–4.86	–4.88	–4.98
	NCI-H460	–7.07	–7.09	–6.85
	A549	–6.01	–6.12	–5.78
	DMS273	–4.88	–4.93	–5.32
	DMS114	–5.05	–4.91	–4.87
**Melanoma**	LOX-IMVI	–6.21	–4.76	–4.84
**Ovarian**	OVCAR-3	–4.88	–4.79	–4.95
	OVCAR-4	–4.77	–4.78	–4.84
	OVCAR-5	–4.92	–4.88	–4.97
	OVCAR-8	–4.75	–4.80	–4.82
	SK-OV-3	–4.88	–4.76	–4.81
**Renal**	RXF-631L	–4.84	–4.75	–4.84
	ACHN	–4.94	–4.90	–4.97
**Stomach**	St-4	–6.24	–5.93	–5.89
	MKN1	–4.87	–4.85	–4.91
	MKN-B	–4.80	–4.92	–5.29
	MKN-A	–4.82	-4.93	–4.95
	MKN45	–4.97	–6.51	–6.13
	MKN74	–4.68	–4.85	–4.85
**Prostate**	DU-145	–4.82	–4.84	–4.85
	PC-3	–4.94	–4.97	–4.95
MG-MID[b]		–5.24	–5.15	–5.18

[a] Kikumori, M.; Yanagita, R. C.; Tokuda, H.; Suzuki, N.; Nagai, H.; Suenaga, K.; Irie, K. *J. Med. Chem.* **2012**, *55*, 5614-5626.

[b] MG-MID: average of the log GI_{50} values of each 39 human cancer cell line.
